# How Chromophore Labels Shape the Structure and Dynamics of a Peptide Hydrogel

**DOI:** 10.1021/acs.biomac.3c01225

**Published:** 2024-01-30

**Authors:** Frederick Heinz, Jonas Proksch, Robert F. Schmidt, Michael Gradzielski, Beate Koksch, Bettina G. Keller

**Affiliations:** † Department of Biology, Chemistry and Pharmacy, Freie Universität Berlin, Arnimallee 22, Berlin 14195, Germany; ‡ Stranski-Laboratorium für Physikalische und Theoretische Chemie, Institut für Chemie, Technische Universität Berlin, Straße des 17. Juni 124, Berlin 10623, Germany

## Abstract

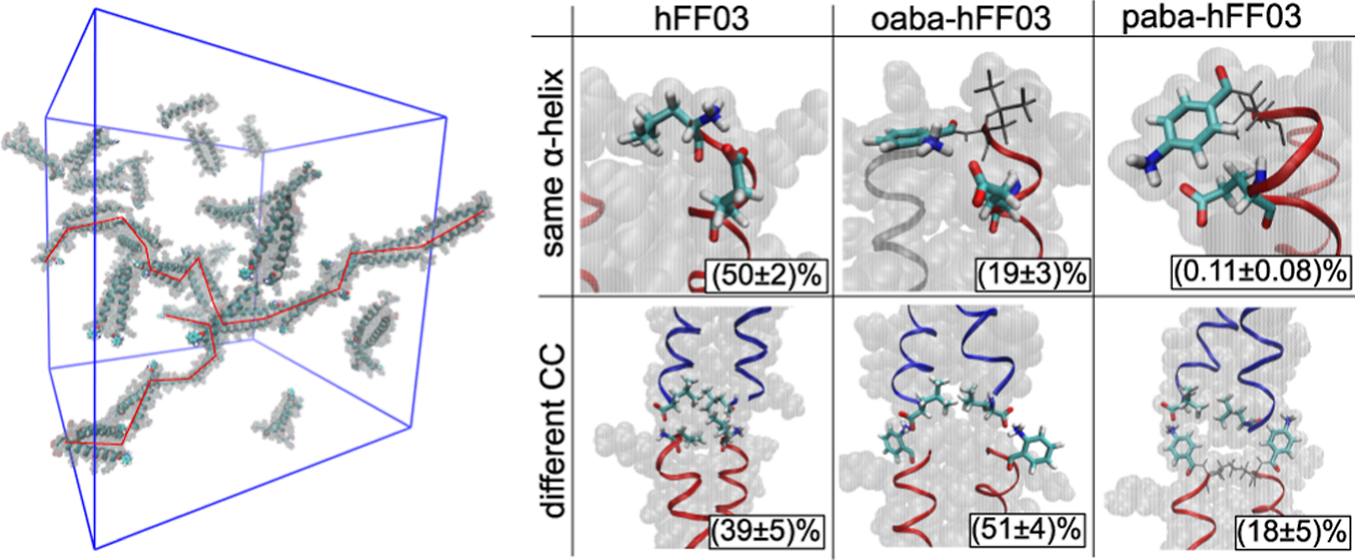

Biocompatible and functionalizable hydrogels have a wide range of (potential) medicinal applications. The hydrogelation process, particularly for systems with very low polymer weight percentages (<1 wt %), remains poorly understood, making it challenging to predict the self-assembly of a given molecular building block into a hydrogel. This severely hinders the rational design of self-assembled hydrogels. In this study, we demonstrate the impact of an N-terminal group on the self-assembly and rheology of the peptide hydrogel hFF03 (hydrogelating, fibril forming peptide 03) using molecular dynamics simulations, oscillatory shear rheology, and circular dichroism spectroscopy. We find that the chromophore and even its specific regioisomers have a significant influence on the microscopic structure and dynamics of the self-assembled fibril, and on the macroscopic mechanical properties. This is because the chromophore influences the possible salt bridges, which form and stabilize the fibril formation. Furthermore, we find that the solvation shell fibrils by itself cannot explain the viscoelasticity of hFF03 hydrogels. Our atomistic model of the hFF03 fibril formation enables a more rational design of these hydrogels. In particular, altering the N-terminal chromophore emerges as a design strategy to tune the mechanic properties of these self-assembled peptide hydrogels.

## 1 Introduction

Hydrogels find diverse biomedical applications,^1^ including drug delivery,^2,3^ tissue engineering,^4^ and wound dressing. Additionally, hydrogels whose viscoelastic properties can be controlled and which can be systematically functionalized can mimic the extracellular matrix^5,6^ or the glycocalyx, i.e., the glycoprotein coat on epithelial cells, and are valuable tools for *in vitro* cell cultures.

Classical hydrogels often consist of long polymers, which are cross-linked by covalent bonds or physical interactions. However, one may also have hydrogels obtained by self-assembly, for instance, consisting of small molecular blocks that self-assemble into fibrils. In that context, peptides are particularly versatile molecular building blocks for self-assembled hydrogels.^7^ Despite the fact that the network structure in self-assembled hydrogels is stabilized by relatively weak hydrophobic or electrostatic contacts, these materials can retain an astonishingly large amount of water. Often more than 99 wt % (mass fraction) of the hydrogel is water.^8^ While the traditional model of intertwined polymer chains can explain water retention^9^ and mechanical properties for a hydrogel with a high polymer mass fraction, this becomes less obvious to rationalize for self-assembled hydrogels with low polymer mass fraction.

Currently, our understanding of the molecular structure of self-assembled hydrogels is limited because many traditional structure analysis methods, such as NMR or X-ray scattering, face challenges in properly resolving the structure of very flexible and dynamic hydrogel networks. The use of small-angle scattering to study such systems has been reviewed recently.^10^ As a result, it is hard to predict whether a given molecule will form a hydrogel or not. Even small and inconspicuous changes to the protein structure or pH value can make or break a hydrogel.^11^ Systematically designing the viscoelastic properties of a hydrogel is currently not feasible because it requires a detailed understanding of the structure and dynamics of the hydrogel network.

Recently, the coiled-coil-based peptide hFF03 (hydrogelating, fibril forming) has been proposed as a scaffold for a functionalizable, biocompatible hydrogel.^12^ hFF03 is a 26-residue peptide, which is designed to self-assemble into α-helical coiled-coil dimers. The dimers are stabilized by a leucine zipper motif and exhibit several solvent-exposed lysine residues to which functional groups, such as carbohydrates, can be attached. hFF03 self-assembles into a 3D fibril network^13^ and has the viscoelastic properties of a hydrogel, even at 0.5 wt % peptide. The viscoelastic properties are close to those of sputum and healthy mucus, and as such, the hydrogel is of special interest for use as a biomimetic hydrogel with medicinal applications. The hydrogel nature of this peptide was verified with the tube inversion test at 4 wt % (4% polymer mass fraction), and the viscoelastic properties were determined by rheological experiments.^13^ Fibril diameter and persistence length were determined by small angle neutron scattering (SANS) experiments.^13^ Furthermore, hFF03 retains its hydrogel character when functionalized with a carbohydrate moiety.^13^

However, the SANS data do not yield any insight into the structure of the fibrils at the molecular level or into the self-assembly mechanism. Two mechanisms are possible for hFF03. First, the coiled-coil dimers could form such that the two peptides are aligned with a zero lateral shift, leading to an aggregation via the charged termini of the coiled-coil dimers. Second, because the sequence of hFF03 consists of three heptad repeat units and a five-residue C-terminal segment, a sticky-end assembly^14^ is conceivable, where the C-terminal segment bridges the gap to the next coiled-coil dimer. In this mechanism, the fibrils are stabilized mainly by a hydrophobic interaction. Because of the bridge between consecutive coiled-coil dimers, this mechanism would immediately explain how long fibrils can arise. We also made the intriguing observation that the presence and isomeric structure of the chromophore label aminobenzoic acid, which was coupled to the hFF03 peptide as a UV–vis marker in ref (13), has a drastic effect on the viscoelastic properties of the substance. It seems plausible that this is a result of the chromophore label interfering with the self-assembly mechanism.

The purpose of this study is to construct an atomistic model of hFF03 that is consistent with the available structural data. Starting from this model, we will conduct MD simulations to elucidate the self-assembly mechanism and to understand how the presence of a chromophore label influences fibril formation. The goal is to identify the crucial microscopic interactions that determine the viscoelastic properties of the hFF03 hydrogels.

## 2 Methods and System

### 2.1 System

**Figure 1:**
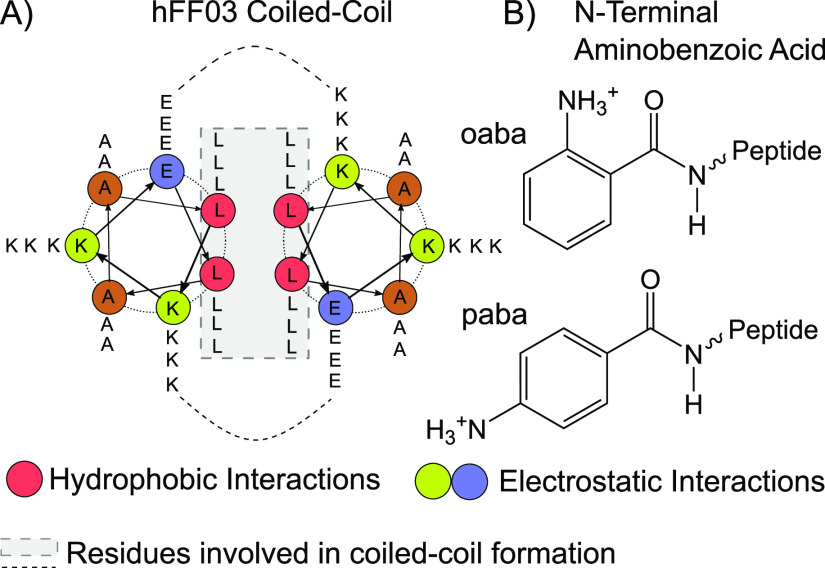
(A) Coiled-coil structure for hFF03 proposed in ref (12). The coiled-coil is stabilized by salt bridges between the polar side chains as well as a hydrophobic core consisting of a leucine zipper motif. (B) N-terminal chromophore aminobenzoic acid. *ortho*-variant oaba at the top and *para*-variant paba at the bottom.

Each hFF03 peptide monomer consists of the sequence *x*-LKKELAA-LKKELAA-LKKELAA-LKKELAA-LKKEL from the N- to C-terminus (Figure 1A). “x” denotes an optional aminobenzoic acid (aba) group (Figure 1B), which is covalently attached to the peptide via a peptide bond between the carboxyl group of aba and the amino group of the peptide N-terminus. Since aba is a chromophore, it allows for the convenient determination of peptide concentration through absorbance measurements. While the experimental concentration of the hydrogel is 0.5 wt % peptide, we use a concentration of 4 wt % in our simulations. This adjustment was made to mitigate computational costs associated with larger box sizes at low concentrations and is a common compromise in studying peptide self-assembly through atomistic simulations.

In ref (13), *ortho*-aminobenzoic acid was used as a chromophore label. We replicate and expand these experiments by synthesizing hFF03 without a chromophore (no-hFF03), hFF03 with *x* = *ortho*-aminobenzoic acid (oaba-hFF03), and hFF03 with *x* = *para*-aminobenzoic acid (paba-hFF03).

### 2.2 Oscillatory Shear Rheology

The rheological measurements were performed on an Anton Paar MCR 502 WESP temperature-controlled rheometer in strain-imposed mode at physiological temperature (37 °C). The temperature was chosen to be in line with earlier rheological experiments on the hFF03 peptide hydrogel in ref (12) and because it is the temperature that is relevant in the context of medical and biological applications. A chromium oxide-coated cone–plate measurement system was used with a diameter of 25 mm, a cone truncation (gap width) of 48 μ m, and a cone angle of 1°. The temperature was set using a Peltier measuring system combined with a Peltier hood to ensure a minimized temperature gradient throughout the sample. To minimize evaporation, a solvent trap was used. The oscillation frequency was varied between 0.05 and 50 Hz at a constant strain amplitude of 5% (a preliminary amplitude sweep showed that this value is still in the linear viscoelastic regime). An up– and down–sweep was performed to check for possible hysteresis effects and the results shown represent averages of both sweeps.

### 2.3 Circular Dichroism Spectroscopy

The oaba-hFF03 peptide was dissolved in Milli-Q water at three different concentrations, and the pH was adjusted to 7.4 with 1 M aqueous NaOH and HCl. The obtained solutions were measured at 37 °C 2 h after preparation by using a Jasco J-810 spectropolarimeter (JASCO Deutschland GmbH, Pfungstadt, Germany) with a Jasco PTC-432S Peltier temperature element. Spectra were recorded using detachable Quartz Suprasil cuvettes with a path length of 0.1 mm (Hellma Analytics, Müllheim, Germany). Spectra are the mean of three measurements and the background is corrected by subtraction of a solvent spectrum.

### 2.4 Molecular-Dynamics Simulations

For the peptide monomers, we use the sequence LKKELAA-LKKELAA-LKKELAA-LKKEL. The initial structures of the coiled-coil dimers are created using the web tool CCBuilder2.0^15^ by Woolfson. Lysine residues and N-terminus are protonated (charge +1), glutamate residues and the C-terminus are deprotonated (charge −1), corresponding to the expected protonation at pH7. For oaba-hFF03 and paba-hFF03, the aminobenzoic acid group was added manually with Pymol^16^ and the force field was calculated with AmberTools,^17^ with the gas charge calculation method. The amino group was protonated. A single coiled-coil dimer has a total charge of +8.

MD simulations were carried out with Gromacs2021+CUDA on the Curta cluster system^18^ with the Amber99SB-ILDN force field.^19^ After energy minimization and relaxation in *NVT* and *NPT* ensemble, the simulations are carried out in the *NPT* ensemble. The temperature was maintained at *T* = 300 K using the velocity-rescale thermostat with a coupling constant of 0.1 ps. The pressure was maintained at 1 bar using a Parinello-Rahman barostat with a coupling constant of 2 ps. The simulation uses a leapfrog integrator with 2 fs per step and periodic boundary conditions in all three spatial direction. Covalent bonds to hydrogen atoms were constrained using the LINCS algorithm. Coordinates were written to the file every 20 ps.

Specific simulation setups are described below. The force field and all input files for the simulations are available via the code repository.

#### 2.4.1 Oligomerisation State

Dimer and tetramer coiled-coil starting structures were created with CCBuilder2.0. Each of these starting structures was solvated in TIP3P water^20^ and the simulation boxes were neutralized with 8 Cl^–^ (dimers) or 16 Cl^–^. After equilibration, the systems were simulated for 100 ns. The diameters were evaluated from the last 50 ns of the simulation, specifically *d*(LEU – C_α_) = distance between opposite leucine C_α_-atoms, *d*(LYS – C_α_) = distance between opposite lysine C_α_-atoms, and *d*(LYS – NH3^+^) = distance between opposite lysine side-chain amino groups, measured at the N-atom. See Figure 3.

**Figure 2:**
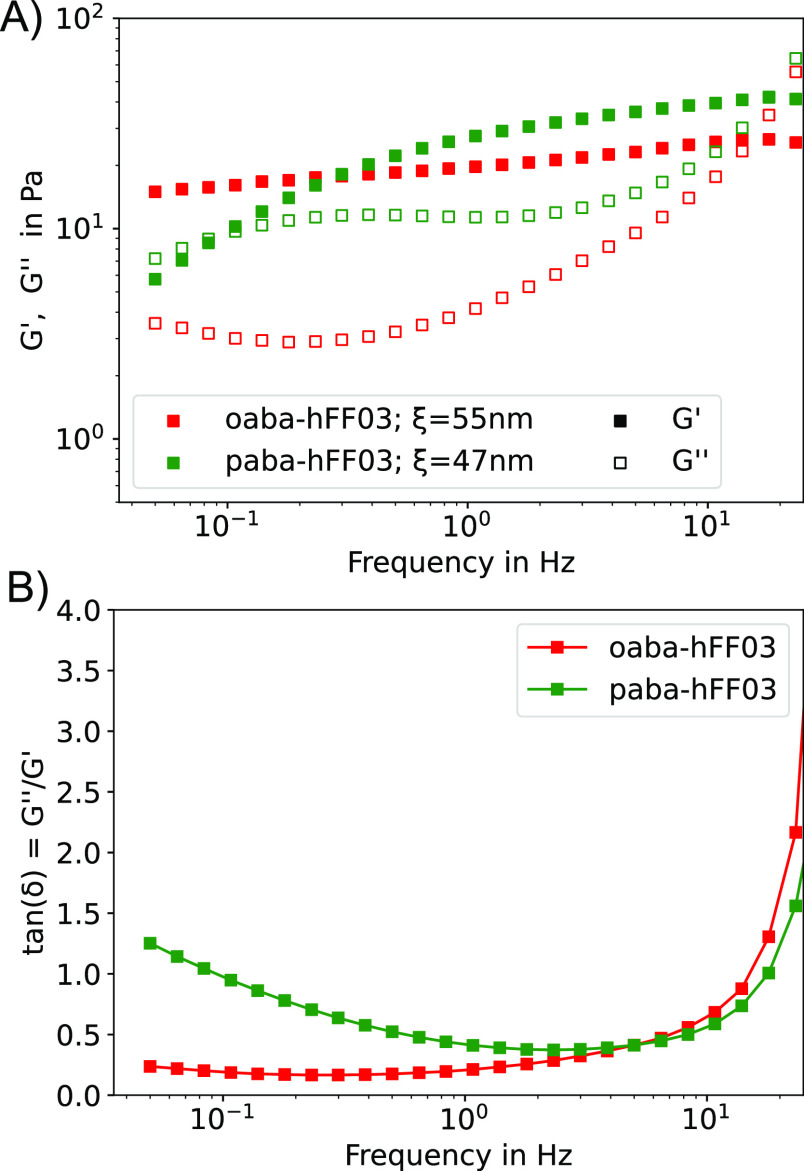
(A) Frequency sweep measured through the oscillatory shear experiment of hFF03 variants. *G*′: storage modulus; *G*″: loss modulus; ξ: mesh size, evaluated at 8.34 Hz. (B) Loss tangent tan (δ) = *G*″/*G*′.

**Figure 3:**
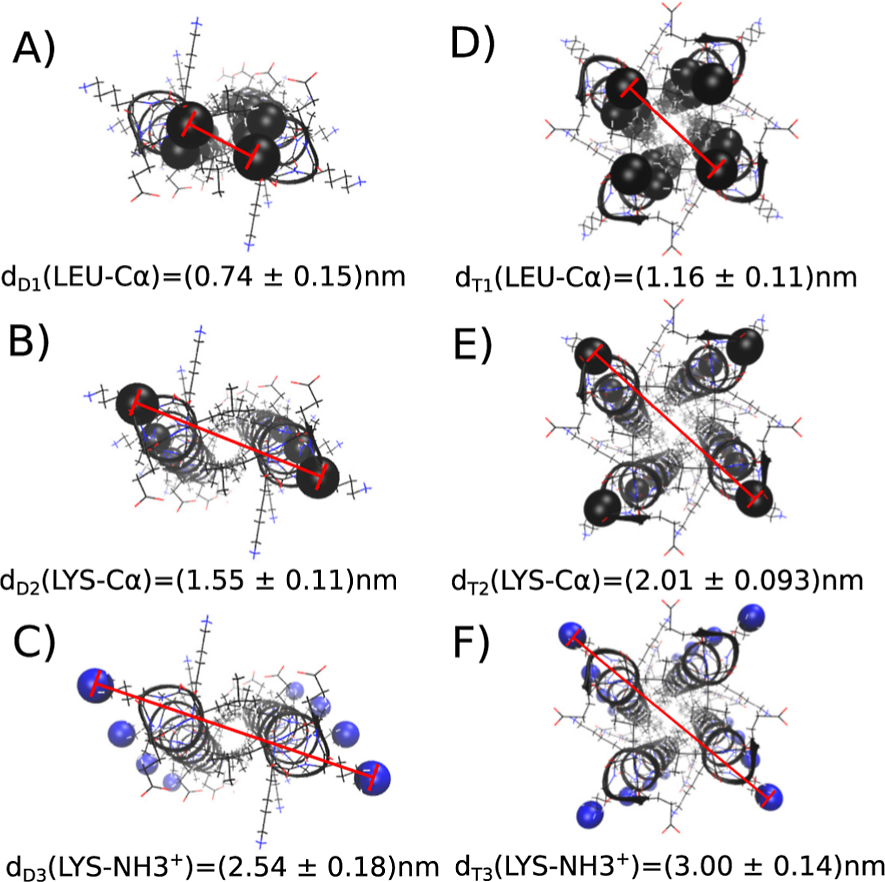
Diameter of coiled-coil dimers (A–C) and coiled-coil tetramers (D–F), averaged from 50 ns MD simulations for each model. Experimental fibril diameter from the SANS experiments ranges from 2.28 to 2.6 nm.^12^

#### 2.4.2 Coiled-Coil Oligomers with Lateral Shift

**Figure 4:**
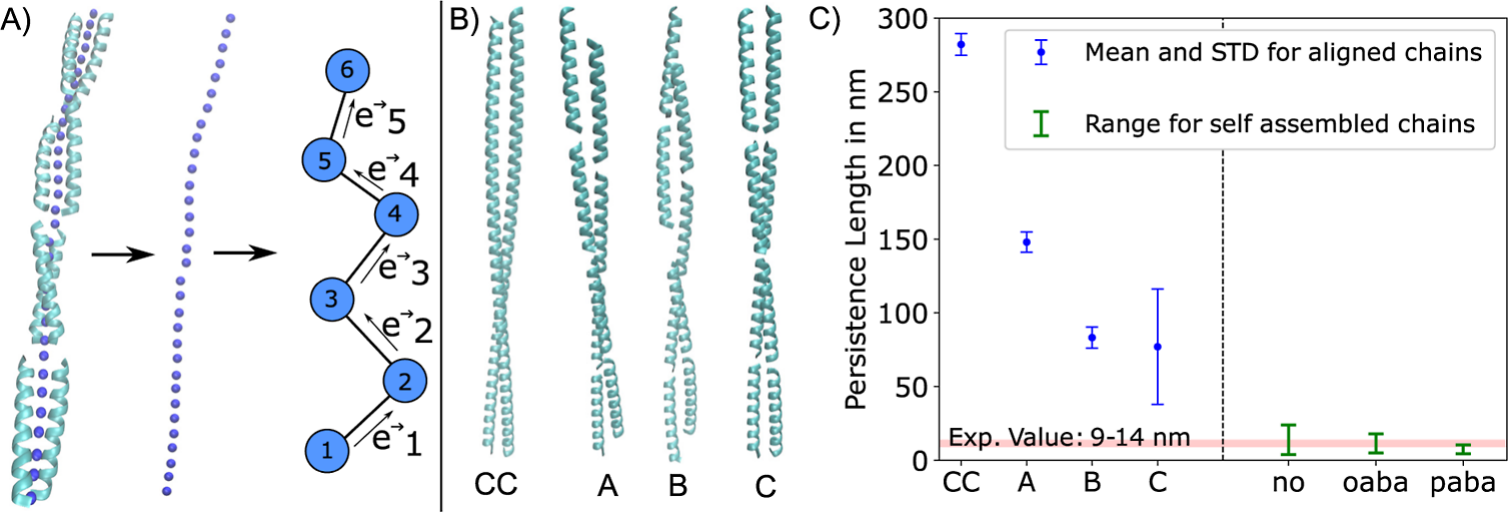
(A) Virtual bead chain at the center of a coiled-coil fibril chain, which we used for the calculation of chain vectors  in eq 2. (B) Models of coiled-coil fibril chains. CC: continuous coiled-coil with LKKELAA heptad repeats. A: lateral shift, but still a continuous leucine zipper. B: small and large lateral shift; the zipper is interrupted at the large lateral shift. C: zero lateral shift, chain of individual coiled-coil dimers. (C) Persistence lengths for different models. CC, A, B, and C are results for aligned fibrils of no-hFF03, and hFF03, oaba-hFF03, and paba-hFF03 for self-assembled fibril chains of type C. Both codes have slightly different chain definitions and cannot be directly compared. The range for self-assembled fibrils is the total range of observed values over all chain lengths with more than 2000 data points. The additionally constrained to a maximum seven coiled coils, since the longer chains behave too erratic. Experimental persistence lengths are from SANS measurements.^12^

A structure of a continuous coiled-coil with 16 LKKELAA heptad repeats was created with CCBuilder2.0,^15^ which served as the starting structure for the model CC in Figure 4. To obtain starting structures for the models A, B, and C, heptad repeat units were cut out from the CC structure and termini were fixed with pdbfixer.^21^ Each starting structure was solvated with TIP3P water^20^ in a rectangular box with 2 nm space around the peptide in all directions. The boxes were neutralized with 32 Cl^–^ anions for the models A, B, and C and 34 Cl^–^ for continuous coiled-coil. Five independent simulations of 150 ns were conducted for each system, but only the last 50 ns were used to calculate the persistence length.

#### 2.4.3 Self-Assembled Coiled-Coils

32 coiled-coil dimers of no-hFF03, oaba-hFF03 or paba-hFF03 were solvated in TiP3P water in a cubic box with 20 nm box length, corresponding to roughly a 4% polymer mass fraction. 256 Cl^–^ anions were added to generate a neutral simulation box. Three independent simulations of 150 ns were conducted for each of the systems.

#### 2.4.4 Local Diffusion Coefficient

A single no-hFF03 coiled-coil dimer was solvated in TIP3P water in a simulation box with size 7 × 7 × 4.7 nm. The box was neutralized with 8 Cl^–^ anions and simulated for 0.2 ns with at simulation time step of 1 fs. Coordinates were written to the file every 10 fs.

The bulk water values for TIP3P were calculated from a simulation of pure TIP3P water with similar setup and simulation time.

### 2.5 Analysis of the MD Simulations

#### 2.5.1 Persistence Length

The persistence length is defined via the following correlation function^22^
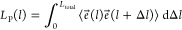
1where ⟨···⟩ represents the ensemble average, *e⃗*(*l*) and *e⃗*(*l* + Δ*l*) are unit vectors of the chain orientation at position *l* and *l* + Δ*l*, and *L*_total_ is the total chain length. Note that *L*_P_(*l*) depends on the initial point *l*. For a chain with *N* discrete beads, spaced at a distance of Δ*l*, eq 1 simplifies to
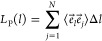
2where  is the local chain orientation at the *j*th bead, and  are the positions of bead *j* and bead *j* + 1. See Figure 4A for sketch.

In principle, the persistence length can be directly calculated from eq 2. However, by construction , eq 2 only yields values *L*_P_(*l*) that are lower than the chain length *L*_total_ = (*N* – 1)Δ*l*. For short and stiff chains, this is unphysical. Under the assumption that a more bent chain is in a higher energy state, it is possible to calculate the expected deformation of a chain with the Maxwell–Boltzmann relation and derive the following equation^22^
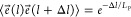
3i.e., the correlation function decays exponentially with the distance from . For each coiled-coil chain in Figure 4, we define a chain of beads with one bead per leucine–leucine zipper pair, where the bead position is the midpoint between the leucine C_α_ atoms. We estimated the left-hand side of eq 3 and fitted an exponential function to obtain *L*_P_.

The persistence lengths in the simulations of self-assembled coiled-coil oligomers were obtained by the same approach, where the oligomer chains were identified by the approach described below.

#### 2.5.2 Identification of Oligomer Chains

Continuous coiled-coil oligomer chains in our simulations of self-assembled coiled-coil oligomers were identified based on salt bridges between coiled-coil dimers. A contact between an ammonium group and a carboxyl group counts as a salt bridge if a hydrogen of the NH _3_^+^ group is within 0.35 nm of either oxygen of the COO^–^ group. We defined a dimer–dimer interaction as a salt bridge between the N-terminus, K2 or K3 of one dimer and the C-terminus or E25 of the neighboring dimer. Our code detects self-assembled chains and in case of a chain split, treats both chains as individual chains. It also detects if the chain binds to itself and forms a circular fibril chain. In this case it stops the chain length and a circular chain is treated as one chain element longer than the sum of its chain elements.

#### 2.5.3 Local Self-Diffusion Coefficient

The diffusion constant *D* of a particle is related to its velocity autocorrelation function (VACF) via
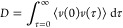
4(Green–Kubo relation), where *v*(0) is the velocity at time *t* = 0 along a single spatial coordinate, *v*(τ) is the velocity at time τ, and ⟨···⟩ denotes an ensemble average. To calculate the VACF ⟨*v*(0)*v*(τ)⟩, we use the Wiener-Kinchin^23^ theorem. This allows for the direct calculation of the VACF from the velocities *v*(*t*) with the help of the fast Fourier transformation algorithm FFT.^24^ The theorem can be summarized such that VACF is the real part of the inverse FFT of the FFT multiplied by its complex conjugate.
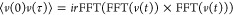
5

To calculate the noncircular autocorrelation function it is necessary to use a so-called zero padding.^25^

To obtain a local water diffusion coefficient dependent on the radial distance to the coiled coil, we use a bead chain, as defined before, to locate the center of the coiled coil. The coiled-coil dimer axis is the vector between the first and last bead of the coiled coil. The radial distance is the distance of the center-of-mass of a water molecule from this axis. The procedure for computing the local diffusion coefficient is outlined in the Supporting Information Section SI.

### 2.6 Code Repository

All code for the analysis of the trajectories and their visualization is written in Julia1.7.3^26^ and is accessible in the code repository with short example files. https://github.com/bkellerlab/hFF03_hydrogels.

## 3 Results

### 3.1 Mechanical Properties of hFF03-Hydrogels

We created probes following the experimental conditions of Hellmund *et al.*(12) and determined their viscoelastic properties using oscillatory shear rheology.

In an oscillatory shear experiment, the material is subjected to an oscillating shear strain, and the resulting shear stress is measured. The ratio of stress and strain yields the complex modulus G* = *G*′ + *iG*″, which is a measure of the material’s overall resistance to deformation. Here, *G*′ is the storage modulus, representing the elastic contribution and *G*″ is the loss modulus, representing the viscous contribution. In a frequency sweep experiment, the oscillation frequency is varied, while the strain amplitude is kept constant. *G*′ and *G*″ are measured as a function of the oscillation frequency. Their values and frequency dependence are characteristic of the linear viscoelastic properties of a material. The tangent of the phase angle (δ) between stress and strain, also known as the loss tangent tan (δ) = *G*″/*G*′, elucidates whether viscous or elastic properties dominate.

In hydrogels, the elastic contribution (storage modulus *G*′) is usually larger than the viscous contribution (loss modulus *G*″), i.e., *G*′ > *G*″, and both moduli are rather insensitive to frequency.^27^ In an ideal system with fibrils that are cross-linked by strong interactions of covalent bonds, both lines would be parallel. But real systems often deviate from this ideal behavior and show more complex behavior.^28^

While it is generally assumed that a chromophore has only little influence on the macroscopic properties of a system, we found that varying the chromophore induced striking differences in the viscoelastic properties of hFF03 (Figure 2). oaba-hFF03 and paba-hFF03 exhibit typical hydrogel behavior, while no-hFF03 shows no such behavior and is a low viscous liquid. The moduli of the hydrogels have values in the range of 5–40 Pa and their behavior is distinctly gel-like, since *G*′ > *G*″. The loss tangent tan (δ), shown on the bottom of Figure 2 is smaller than 1 over nearly the whole frequency range, indicating predominantly gel-like behavior. Their gel character is also corroborated by the tube inversion test. While both isomers exhibit gel-like behavior, oaba-hFF03 has a significantly smaller loss tangent, making it the stronger hydrogel of the two. In contrast, for paba-hFF03 *G*′ becomes substantially smaller and the loss tangent becomes larger at lower frequency, which means that its structural relaxation time is much shorter and it will relax mechanical stress after much shorter times than oaba-hFF03.

We can use the value of *G*′ in the plateau region, *G*_0_, to give an estimate for the characteristic size ξ, i.e., the mesh size of the hydrogel network.^29−31^ Assuming a mesh of size ξ, where each mesh stores an energy of *k*_B_*T*, one arrives at the relation: . For the calculation of ξ, we take *G*_0_ to be the value of *G*′ at a frequency of 8.34 Hz. Using *G*_0_, we find mesh sizes of around 55 and 48 nm for oaba-hFF03 and paba-hFF03, respectively. Since no true plateau is seen for *G*′, the chosen frequency is rather arbitrary and the values for ξ can be regarded as an upper estimate.

The viscoelasticity of no-hFF03 was too low to be accurately measured at higher frequencies and we present the results in the Supporting Information (Figure S1) solely for the sake of completeness. We conclude that the chromophore is not an “innocent” label. Its presence is critical for hydrogel formation in hFF03, and even the position of the amino group in the chromophore influences the stability and mechanical properties of the hydrogel. To better understand this behavior, we set out to construct an atomistic model of self-assembled hFF03 peptides.

### 3.2 Structural Model

Previous SANS and cryogenic transmission electron microscopy (Cryo-TEM) showed that oaba-hFF03 self-assembles into fibrils with a diameter of 2.28–2.60 nm and a persistence length of 9 to 14 nm.^12^ Because of the design of the peptide sequence,^32,33^ we can assume that the peptides form α-helices which have a hydrophobic flank consisting of leucine residues. The peptides can self-assemble into coiled coils via this hydrophobic region (leucine zipper motif). For the atomistic model, we need to consider the following aspects: (i) oligomerization state (How many peptide strands are assembled across the diameter of the fibril?); (ii) orientation the helices within the fibril (do all peptides have the same N-to-C-terminus orientation along the fibril?), and (iii) lateral shift of the α-helices within respect to each other.

#### 3.2.1 Oligomerization State and Helix Orientation

The oligomerization state is the number of peptide helices that self-assemble into a coiled-coil oligomer and the relative orientation of these helices. hFF03 is designed following the rules of coiled–coiled building formulated by Woolfson,^34^ and thus it is highly likely that hFF03 self-assembles into a parallel coiled-coil dimer. Sequences can be tested for their preferred oligomerization state using the tool LOGICOIL.^35^ For hFF03, the preference is for a parallel dimer with 0% chance of forming a trimer. This structure of a parallel dimer has also been proposed by Hellmund *et al.*(12)

We used MD simulations to verify this model and simulated different oligomerization states and helix orientations of no-hFF03. The models were constructed without a lateral shift, and we assume that the chromophore has no influence on the oligomerization state.

Coiled-coils modeled as antiparallel dimers were not stable and drifted apart in the simulation. By contrast, coiled-coil models as the parallel dimer and parallel tetramer remained stable during 50 ns MD simulation. In the tetramer model, the leucine flanks of the four α-helices form a joint hydrophobic core. By contrast, modeling a tetramer as two parallel dimers next to each other did not yield a stable complex.

Figure 3 compares the diameter of the coiled coils, measured at different reference points, to the experimental diameter.^12^ Taking the amino groups of opposing lysine side chains as reference points (Figure 3C,F) likely best represent the SANS experiment. The diameter of the parallel dimer model (Figure 3C) matches the experimental results of 2.28 to 2.60 nm,^12^ while the diameter of the tetramer dimer model (Figure 3C) is too large. Thus, the parallel dimer coiled coils are consistent with the SANS experiment as well as the prediction by LOGICOIL,^35^ and we used this model in all further simulations.

#### 3.2.2 Lateral Shift and Persistence Length

Next, we discuss the lateral alignment of the two parallel α-helices within the coiled-coil fibril. Because of the repeated motif LKKELAA in the peptide sequence of hFF03, α-helices could self-assemble such that the termini do not line up, but are shifted by one or two repeat motifs (lateral shift, sticky-end assembly).^14^ hFF03 does not feature an anchor, such as a disulfide bridge, that could enforce a particular lateral shift. Sequences, for which lateral shift and overlapping helices have been reported, either have oligomerization states of five or more helices,^36^ or consist of helices with different charges at either end.^37^

Importantly, fibrils with and without lateral shift self-assemble via different interfaces^38^ and would result in different fibril flexibility. With no lateral shift, each dimer can move independently of the other dimers, and elongated fibrils arise if the dimers align linearly, possibly stabilized by salt-bridges and hydrogen bonds between the N- and C-termini of adjacent dimers (structure C in Figure 4.). This would lead to very flexible fibrils, and it is unclear whether the resulting aggregate would be sufficiently stable to explain the observed viscoelasticity. By contrast, with a lateral shift of one or two repeat motifs, one α-helix would bridge the gap to the next coiled coil via the leucine zipper motif. The fibril chain is then stabilized by the same hydrophobic contacts that stabilize the coiled coil (structures A and B in Figure 4). Because of the overlap, we expect a stiffer fibril than in the aggregate with zero overlap.

To test this, we measured the fibril chain flexibility in models with different lateral shifts and compared the computational values to the results of SANS experiments.^12^ Only structures with no or small lateral shifts were stable in our simulations (structures A, B, and C in Figure 4B). We also include a continuous coiled coil with the same peptide sequence for comparison (structure CC in Figure 4B).

The persistence length *L*_P_, or chain decorrelation length, is a measure for the flexibility of a chain.^22,39^ When *l* is the position along the chain, one compares the local chain orientation at *l*, *e⃗*(*l*), to the chain orientation *e⃗*(*l* + Δ*l*) at Δ*l* further down the chain. *e⃗*(*l*) and *e⃗*(*l* + Δ*l*) are unit vectors. *L*_P_ is the length at which the chain orientation at *l* + Δ*l* is fully uncorrelated from the chain orientation at *l* (see Section 2).

To calculate *L*_P_ we discretized the coiled-coil chain. A discretization along the peptide backbone (as preimplemented in programs that calculate peptide chain flexibility) returns the flexibility of the α-helix within the coiled-coil, not the flexibility of the coiled-coil fibril chain. Instead we define a new virtual chain of beads following the core of the coiled-coil, shown in Figure 4A. We define a bead for each leucine–leucine zipper pair, where the bead position is the midpoint between the leucine C_α_ atoms, which places the beads at the center of the coiled-coil.

Figure 4C compares the persistence lengths of our model systems to the experimental persistence length of oaba-hFF03 of 9 to 14 nm.^12^ The continuous coiled-coil (CC), is very stiff with a persistence length of roughly 290 nm, which is in line with previously reported persistence lengths of peptide coiled-coils.^39^ Interrupting the chain and introducing lateral shifts leads to more flexible fibril chains (A, B, and C). However, systems with overlapping peptide strands have persistence lengths that are much larger than the experimental value. System C, in which we manually aligned coiled-coil dimers with zero lateral shift into a straight fibril, is still stiffer than the experimental value, but the estimate shows a large standard deviation.

Closer inspection of the simulations showed that the fibril chain started to fluctuate, leading to kinks at the interface between adjacent coiled-coil dimers, causing the large standard deviation. More specifically, the starting structure of the coiled-coil-dimer fibril was cut from a continuous coiled-coil with the same heptad repeat unit, generating a very tight dimer–dimer interface. In half of the simulations, these interfaces loosened. This also implies that the manually aligned, very straight fibril chain conformation is not at a free-energy minimum and the conformational equilibrium is more dynamic. To test this, we simulated randomly placed coiled-coil dimers of no-hFF03 in water. We observed that these coiled-coil dimers rapidly self-assemble into fibril chains within nanoseconds. Occasionally, fibril chains self-interact across the periodic boundary of the simulation box. However, these interactions are short-lived because these fibrils are highly dynamic and continuously break and reassemble. We do not expect that this self-interaction across the periodic boundary significantly over stabilize the fibril chains. We repeated these simulations with oaba-hFF03 and paba-hFF03, which showed the same behavior. The persistence lengths of these self-assembled fibril chains are shown as green ranges in Figure 4C. They agree well with the experimental value.

We conclude from Figure 4 that coiled-coil assemblies with nonzero lateral shifts are too stiff compared to the experimental persistence length. Therefore, fibril formation via the leucine zipper motif can be ruled out. Instead, coiled-coil dimers with zero later shift (model C) self-assemble into highly dynamic fibril chains with a persistence length of about 10 nm. These fibrils are likely stabilized by hydrogen bonds and salt bridges between the N- and C-termini of adjacent coiled coils.

**Figure 5:**
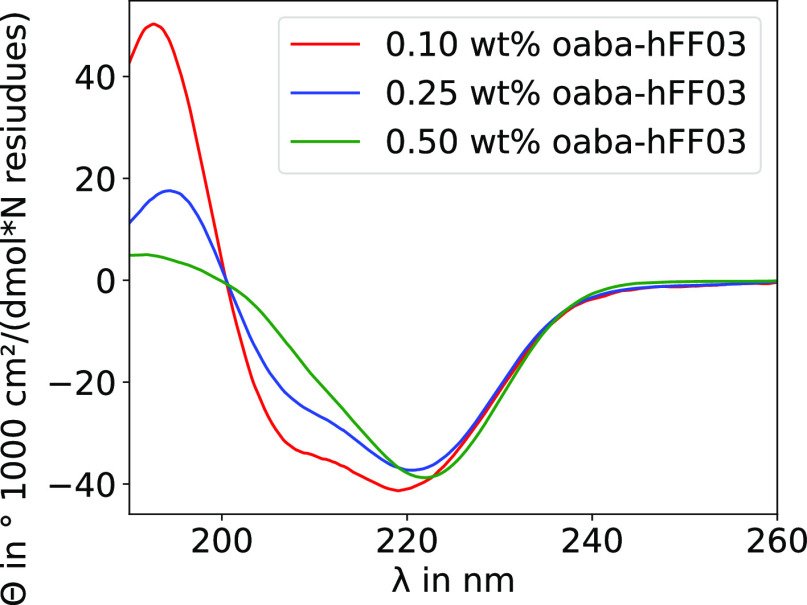
Circular dichroism spectra oaba-hFF03 in water at pH 7.4, *T* = 37 °C, and concentration in mass percentage. The 222 nm/208 nm ellipticity ratios are 1.19 at 0.1 wt %, 1.55 at 0.25 wt %, and 2.66 at 0.5 wt %.

This two-step mechanism (coiled-coil formation, followed by self-assembly into fibrils) is supported by circular dichroism (CD) spectroscopy (Figure 5). At low concentrations, the characteristic signals for α-helical structures, namely, an ellipticity maximum at 195 nm and two minima at 208 and 222 nm, respectively, are clearly present. The ellipticity minimum at 222 nm is of higher intensity than the minimum at 208 nm, indicating the formation of more ordered structures.^14^ While it is possible to calculate the percentage of coiled-coils,^40^ this would not change the interpretation of the result. The intensity ratio (or ellipticity ratio) of these two minima increases as the concentration is increased, indicating that at higher concentrations, more highly ordered structures are formed. A possible mechanistic interpretation is that at low concentrations individual coiled-coils are present, which only self-assemble into fibrils if a certain concentration threshold is passed.

### 3.3 Influence of the Chromophore on the Fibril Stability

**Figure 6:**
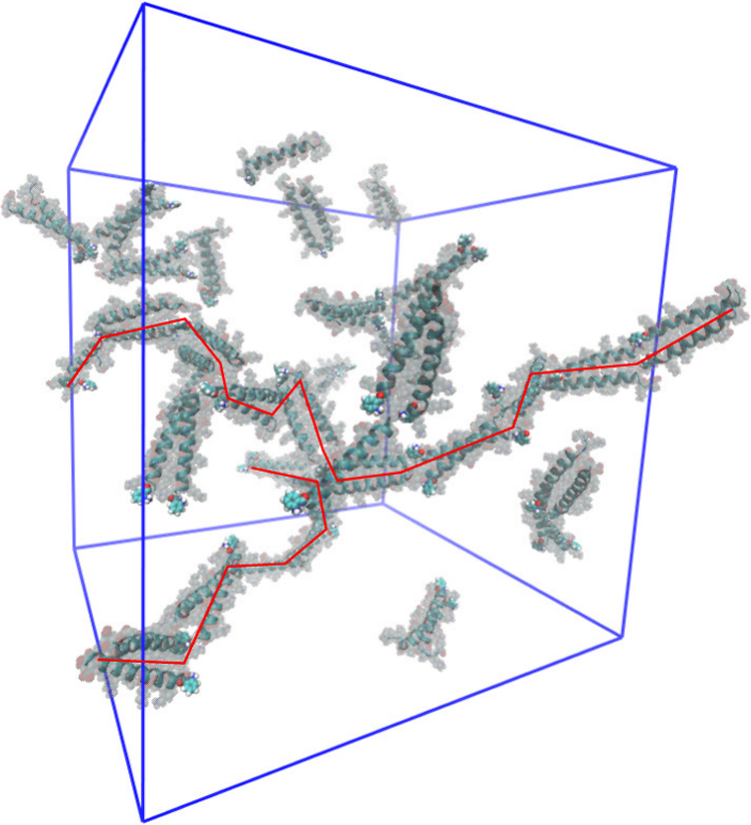
Snapshot of oaba-hFF03 in explicit water after 50 ns simulation (4 wt %, starting structure with randomly placed coiled coils). Red lines highlight self-assembled oligomers.

Next, we investigated the influence of the chromophore on the structure and stability of the self-assembled fibril chains. For all three systems, we simulated 32 randomly placed coiled-coil dimers in a water box of 20 × 20 × 20 nm^3^ for 150 ns. For each system, three separate simulations with random start configurations were performed. These systems correspond to a polymer mass fraction of 4 wt %. A snapshot of the oaba-hFF03 simulation after 50 ns is shown in Figure 6 (see Figure S2 for a snapshot of no-hFF03 and paba-hFF03).

**Figure 7:**
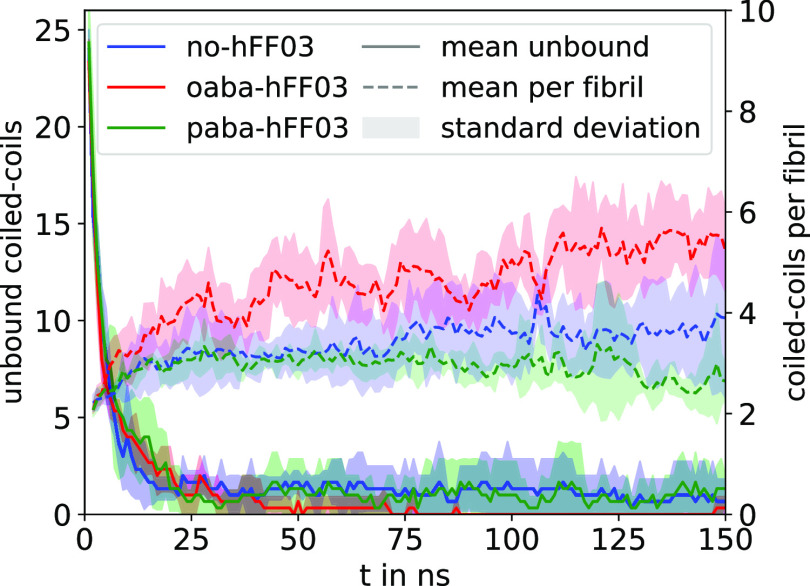
Time series of the number of unbound coiled-coil dimers and the number of coiled-coil dimers per oligomer chain. For each system, we conducted three MD simulations. Average and standard deviation were calculated as running averages with a block size of 1 ns and then averaged over the three separate trajectories for each system.

The coiled-coil dimers rapidly form oligomers during the first few nanoseconds. These oligomer chains then elongate. The elongated oligomers are highly dynamic and keep rearranging on a time scale of nanoseconds. The progress of the oligomer formation is illustrated in Figure 7. The number of coiled-coil dimers, which are not part of an oligomer (solid lines), rapidly falls in the first few nanoseconds and reaches a plateau after about 50 ns. While for no-hFF03 and paba-hFF03 we find 1–2 unbound coiled-coil dimers per simulation box, all coiled-coil dimers in the oaba-hFF03 simulations are bound in oligomers. As the number of unbound coiled-coils decreases, the average size of the oligomers increases (dashed lines). It reaches a plateau of 2 to 3 coiled-coils per oligomer for paba-hFF03. In no-hFF03 and oaba-hFF03, we observe a slight drift to longer oligomers throughout the simulation.

**Figure 8:**
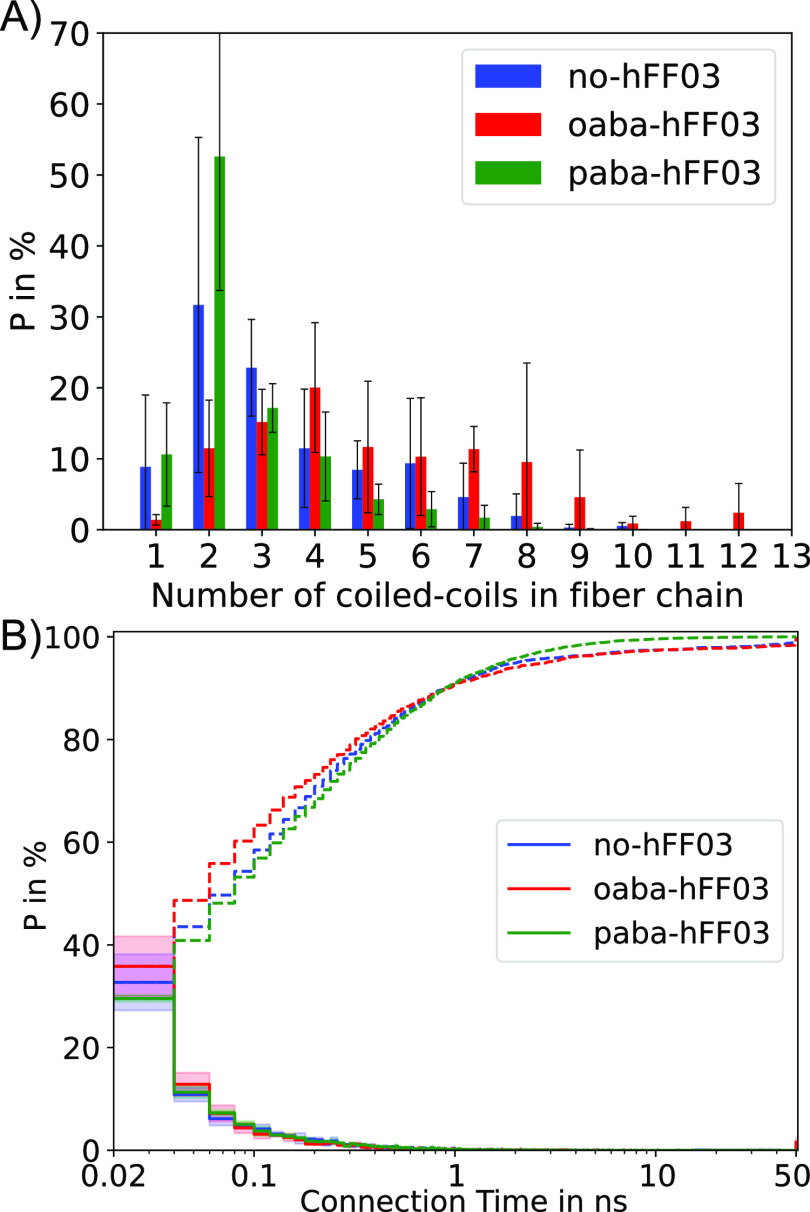
(A) Oligomer size distribution between 100 and 150 ns of the MD simulation, averaged over three independent simulations for each system. Error bars indicate the standard deviation. (B) Residence time distribution of the coiled-coil dimer interactions (solid line) and corresponding cumulative distribution (dashed line) between 100 and 150 ns of the MD simulation. oaba-hFF03 exhibits coiled-coil dimer interactions that do not break in this time interval.

We use the last 50 ns of the simulations to extract statistics on the oligomer sizes (Figure 8A) and lifetimes (Figure 8B). Since the coiled-coil dimer interaction reassembles on the time scale of 1 ns, i.e., much shorter than the sampling time of 50 ns, we do not expect that overstabilization due to the periodic boundary conditions in the simulation distorts the analysis. The oligomer size distribution (Figure 8A) shows sizable statistical uncertainties, which indicates that simulations are not fully converged, yet. Nonetheless, some trends are evident. With a maximum probability at four coiled-coil dimers per oligomer, oaba-hFF03 forms longer oligomer chains than the other two systems, whose distributions peak at two coiled-coils dimers per oligomer. Additionally, oaba-hFF03 forms the longest chains with up to 12 coiled-coils dimers per oligomer.

Figure 8B shows the lifetime distribution of the coiled-coil interactions. Since the coiled-coils rearrange on the nanosecond time scale, the statistical uncertainty in this distribution is much smaller than in Figure 8A. The fast dynamics are also evident from the fast decay of the lifetime distribution (solid line): most coiled-coil dimer interactions only last a few 100 picosecond, and almost all of them are broken within the first nanosecond.

The dashed lines show the cumulative distributions in Figure 8B, which reveal differences between the systems on longer time scales. (note the logarithmic scale on the time axis). For paba-hFF03, the cumulative distribution reaches 100% within a few nanoseconds, indicating that within a time window of 10 ns every coiled-coil dimer interaction in our simulation is broken. By contrast, for oaba-hFF03 we find coiled-coil dimer interactions lasting 50 ns and longer. On may speculate that these long-lived oligomers serve as a nucleus that initializes the formation of fibrils on time scales beyond the time scale of our simulation. This might explain why oaba-hFF03 forms a stronger hydrogel than paba-hFF03. Interestingly, no-hFF03, which does not form a hydrogel, also exhibits long-lived coiled-coil dimer interactions. It is important to point out that the disruption of an individual coiled-coil dimer interaction does not mean that the oligomer chain falls apart. Rather, the two stubs quickly reassemble with the same oligomers or other nearby oligomers into new oligomer chains. Differences in the viscoelastic properties of the three-peptide systems likely arise from an interplay between the average fibril length and the reassembly rate between fibrils.

It is of note that unlike previously reported N-terminal aromatic modifications^41^ we can not observe any π stacking in our simulations.

Overall, these data show that the presence of the aba chromophore influences the size and stability of the coiled-coil oligomers. Strikingly, even the position of the amino group in the aba chromophore influences the size and stability of the coiled-coil oligomers.

### 3.4 Structural Analysis of the Coiled-Coil Interface

**Figure 9:**
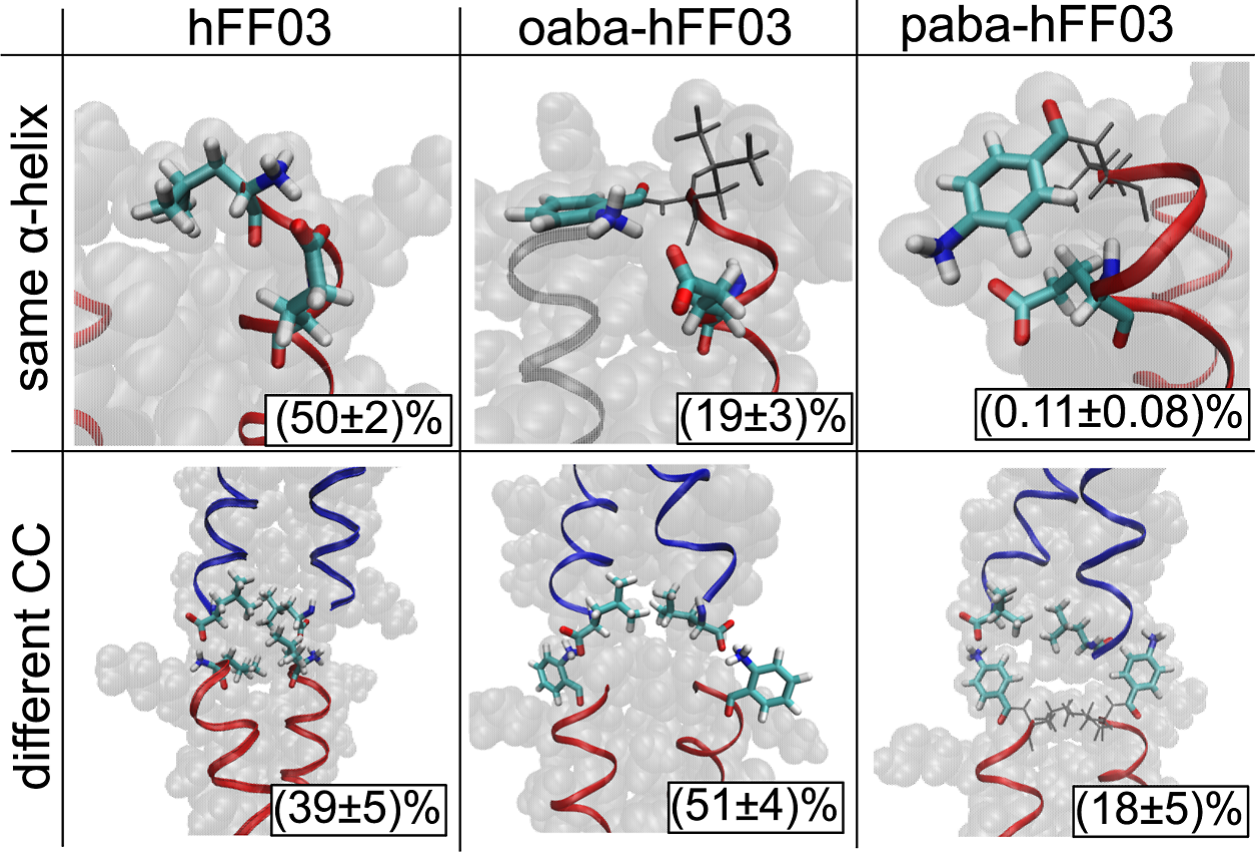
Influence of the chromophore label aba on the coiled-coil dimer interaction. Upper row: salt bridges of the N-terminus within the same α-helix; lower row: salt bridges of the N-terminus to another coiled-coil dimer.

To elucidate the structural origin for the variation in the oligomer sizes and lifetimes, we analyzed the hydrogen bonds and salt bridges in the coiled-coil dimer interface. In no-hFF03, the interface between two coiled coils is predominantly stabilized by a salt bridge between the positively charged amino group of the N-terminus and the negatively charged carboxyl group of the C-terminus (Figure 9). Additionally, the nearby lysine K2 can take the role of the N-terminus, and the nearby glutamic acid E25 in the other coil–coil can take the role of the C-terminus, such that in total the interface is stabilized by several fluctuating salt bridges.

In oaba-hFF03 and paba-hFF03, the aba-group is covalently attached to the N-terminus, and the amino group of the aba-group replaces the N-terminal amino group in the interface. We model the amino group in aba as protonated and positively charged and observe salt bridges from the amino group to the C-terminus and the glutamic acid in the adjacent coiled coil. The salt bridge between coiled-coil dimers competes with an intramolecular hydrogen bond (i.e., within the same peptide chain) between the protonated amino group (N-terminus or aba) and the carboxyl group of the nearest glutamic acid: E4 (Figure 9).

While we observe the same types of salt bridges in all three systems, the relative populations vary drastically across the systems. (See Figures S3–S5 for all salt bridges.). In no-hFF03, the protonated amino group forms an intramolecular salt bridge in 50% of all frames and a salt bridge to an adjacent coiled coil in 39% of all frames. Interactions with the surrounding water are observed in only 11% of all frames. In oaba-hFF03, this equilibrium shifts in favor of the intercoiled-coil salt bridge, which is now populated to 51%. The population of the intramolecular salt bridge is decreased to 19%, and interactions with water slightly go up to 30%. In paba-hFF03, the situation is quite different from those of the other two systems. The amino group most frequently interacts with the surrounding water and engages only in 18% of all frames in an intercoiled-coil salt bridge. The intramolecular salt bridge is almost never populated (0.1%) see Table 1.

**Table 1:** Salt Bridge Population of the N-Terminal Amino Group^a^

	N-terminal salt bridge to/in %			
	same α-helix	same CC	different CC	Solvent
no-hFF03	50 ± 2	0.7 ± 0.3	39 ± 5	11 ± 7
oaba-hFF03	19 ± 3	0.8 ± 0.5	51 ± 4	30 ± 6
paba-hFF03	0.11 ± 0.08	0.14 ± 0.12	18 ± 5	82 ± 5

a See Figure 9 for examples of the different salt bridges. Populations are calculated over all salt bridges in a given category and all α-helices in the simulation box. Mean and standard deviations are calculated from the three independent simulations for each system.

The steric arrangement of the salt bridge seems to be the cause of this shift in the salt-bridge populations. By moving the protonated amino group from the C-terminus to the abachromophore, one moves it away from the carboxyl group of E4, thus weakening the intramolecular salt bridge. Because in oaba, the amino group is in *ortho*-position the oaba-group can be oriented such that the amino group points toward the E4. This is not possible if the amino group is in the *para*-position, and hence the intramolecular salt bridge is not formed in paba-hFF03. On the other hand, moving the protonated amino group to the aba group makes the coiled-coil interface less crowded than in no-hFF03. Figure 9 shows that in oaba-hFF03 the salt bridge can turn outside toward the solvent, leaving enough space for the two L1 and the two L26 residues to orient themselves in the hydrophobic center of the interface. Finally, in paba-hFF03, the protonated amino group points toward the solvent, and it is difficult to find a conformation in which both salt bridges are formed across the coiled-coil interface.

Two other effects contribute to the stability of the coiled-coil interface. First, as mentioned above, the salt bridge between the protonated amino group and the deprotonated carboxyl group of the C-terminus in the adjacent coiled coil can be replaced with salt bridges involving K2 and E25. However, the stability of these salt bridges follows the same trend as that of the dominant salt bridge. Second, one can speculate that hydrophobic effects play a role. The aba-group certainly makes the N-terminus more hydrophobic. On the other hand, the charges at the N-terminus of no-hFF03 are often capped by the intramolecular salt bridge, which also generates a relatively hydrophobic N-terminus. Quantifying these hydrophobic effects is difficult.

In summary, the steric ease with which the salt bridge between the protonated amino groups at the N-terminal ends of one coiled coil and the C-terminal carboxyl group at the adjacent coiled coil can be formed determines how stable the coiled-coil interface is. The stability of this salt bridge (Figure 9) is directly correlated to the oligomer size distribution of the three substances (Figure 8A). However, the stability of the salt bridge and the oligomer size distribution (on the time scale of our simulations) do not explain the differences in the mechanical properties of the hFF03-hydrogels (Figure 2).

### 3.5 Water Retention in the hFF03 Hydrogels

To gain a better understanding of how the coiled-coils influence the structure and dynamics of the nearby water molecules, we analyzed the water density and local diffusion constant in the hydration shell of a no-hFF03 coiled coil.

The self-diffusion coefficient of a particle *D* is a transport property that is usually calculated as the slope of the mean-square displacement versus time. Because the particle is allowed to diffuse away from its initial position during this calculation, this estimator is not suited to calculate local self-diffusion constants. Instead, one can use the Green–Kubo relations^42^ to express this transport property as the particle’s VACF
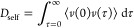
6where *v*(0) is the particle’s velocity at time *t* = 0, *v*(τ) is its velocity at time *t* = τ, and the VACF ⟨*v*(0)*v*(τ)⟩ is the ensemble average of *v*(0)*v*(τ). The ensemble averages for different lag times τ are integrated from τ = 0 to τ = ∞ to obtain the diffusion constant. Of course, for long lag times, τ, the particle diffuses away from its initial position. But in water, the VACF levels off to zero at around 800 fs, and the integral converges at an integration limit of a few picoseconds. This is shorter than the time scale of structural rearrangements in the solvation shell, which usually takes tens of picoseconds. Thus, eq 6 indeed allows us to define a spatially resolved diffusion constant. Equation 6 is calculated using the fast Fourier transformation algorithm. See Section 2. Note that the self-diffusion coefficient of bulk TIP3P water of *D*_self_ = 6.78 × 10^–5^ cm^2^/s obtained from our calculations is slightly higher than previously reported values calculated using the mean-square displacement^43,44^ with values around *D*_self_ = 5.8 × 10^–5^ cm^2^/s. However, the water density is also slightly lower for our simulations. While it is known that TiP3P water has a higher self-diffusion coefficient than the experimentally obtained value of *D*_self_ = 2.3 × 10^–5^ cm^2^/s at 300 K this should not detract from the results.^45^

**Figure 10:**
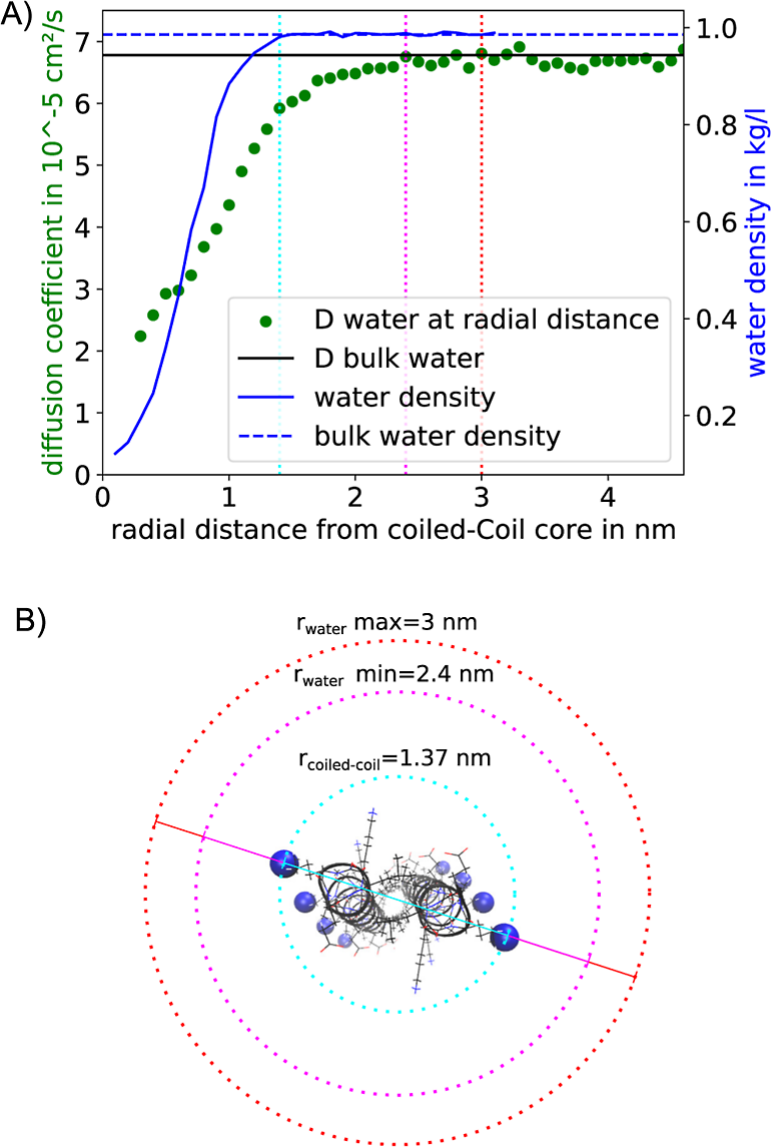
(A) Local density and diffusion constant of water in the vicinity of hFF03. The distance is the radial distance of the water molecule to the center of the coiled coil. The bulk values were calculated from the MD simulation box of pure water. Vertical lines show radii at which bulk properties are restored: density (cyan), diffusion constant, lower boundary (pink), diffusion constant, and upper boundary (red). (B) Visualization of the radii around a coiled-coil dimer. The radius at which the density of bulk water is reached coincides with the coiled-coil dimer radius (cyan).

The solid blue line is in Figure 10. A shows the local water density around the coiled-coil dimer. It increases from zero at the center of the coiled coil to the density of bulk water. The density curve levels off at *r* ≈ 1.37 nm, which is very close to the coiled-coil radius that we determined in Figure 3. Because water molecules can penetrate between the side chains, the water density does not immediately drop to zero at this radius but slowly decreases.

However, the influence of the coiled-coil dimer reaches beyond *r* ≈ 1.37 nm. This can be seen from the local diffusion constant, which only reaches the bulk diffusivity at between *r* = 2.4 nm and *r* = 3.0 nm. That is, within the solvation shell from 1.4 to 2.4 nm, rearrangements of the water structure are slower than in bulk water.

By comparing the volume of the coiled-coil dimer including its hydration shell to the volume of the simulation box, we can calculate how much bulk water remains in the system. We approximate the shape of the coiled-coil dimer including its solvation shell as a cylinder whose volume is *V*_cylinder_ = π*r*^2^*h*, where *r* is the radius of the cylinder and *h* is its height. We set *r* = 2.4 nm (radius of the hydration shell) and *h* = 4.0 nm (length of the no-hFF03 coiled-coil dimer), which yields a volume of *V*_cc_ = 72.4 nm^3^. Our simulation box has a volume of *V*_box_ = 20 × 20 × 20 nm^3^ = 8000 nm^3^ At a concentration of 4 wt %, it contains 32 coiled-coil dimers. Thus, the volume fraction of bulk water is 1–32·*V*_cc_/*V*_box_ = 0.710, i.e., 71%. If we assume that the hydration shell reaches *r* = 3.0 nm, the bulk water content decreases to 55%. In either case, at these high concentrations, a hFF03-hydrogel consists to a substantial part of coiled-coil peptides and water bound to these peptides.

However, Figure 5 shows that fibril formation sets in at much lower concentrations. In Figure 2, the mechanical properties of the hFF03-hydrogel have been measured at 0.5 wt %. At this concentration, the bulk water content ranges between 96% (for *r* = 2.4 nm) and 94% (for *r* = 3.0 nm). That is, almost everywhere in the sample, the water moves unhindered. Retention of water close to the coiled-coil fibrils therefore seems to contribute little, if any, to the observed hydrogelation.

## 4 Conclusions

We developed an atomistic model of self-assembled peptide hydrogel hFF03. The peptides within this structure form α-helical coiled coils with zero lateral shift. These coiled-coil dimers self-assemble into oligomers on the nanosecond time scale by forming salt bridges between the C and N-terminus of neighboring coiled-coil dimers. Our model aligns well with the previously published small-angle neutron scattering (SANS) data and circular dichroism (CD) spectra presented in this study. Specifically, the oligomers match the experimental diameters and persistence lengths. Our model refutes the possibility of a sticky-end assembly, where hydrophobic contacts between leucine residues stabilize the oligomers.

The chromophore aminobenzoic acid, which was originally added at the N-terminus as a UV–vis marker, has a significant impact on the structure and dynamics of the coiled-coil dimer interface. Altering the position of the amino group from the ortho- to para-position in aba shifts the equilibrium between salt bridges and hydrogen bonds with the surrounding water, subsequently influencing the sizes and stability of the oligomers.

The striking consequence of this is that the chromophore label controls the rheological properties of the hFF03 hydrogels. In fact, the presence of the chromophore label is crucial for hydrogel formation as no-hFF03 does not form a hydrogel. oaba-hFF03, which generates the longest and most stable oligomers of the three variants, also forms the most stable hydrogel at the macroscopic level. In contrast, paba-hFF03 forms shorter oligomers and also a softer gel with a markedly shorter structural relaxation time.

Our model does not fully account for the macroscopic results, and especially, the oligomer size distribution does not fully correlate with the rheological data. How long-range interactions necessary for the elastic component of the viscoelastic properties arise from the rapid coiled-coil rearrangements observed in our simulations is not yet explained.

The largest difference between simulations and oscillatory shear experiments is the time scale. With an aggregated simulation time of 450 ns per system, we have probed the dynamics of hFF03-hydrogels on time scales from 10^–12^ to 10^–7^ s (THz to 0.1 MHz). By contrast, the oscillatory shear experiments probed the dynamics on time scales from 10^–2^ to 10 s (10 mHz to 10 Hz), i.e., they probe the structural relaxation time of the complete system. In contrast, the simulations only probe the first basic elementary step involved in the process of gelation, which is the coiled-coil dimer interaction. The whole rheological relaxation process is, of course, that of the whole system of many such cross-links. In that respect the situation is similar to that of hydrophobically cross-linked hydrogels, where the individual hydrophobic sticker has a lifetime of μ s while the structural relaxation time will be in the range of many seconds, both scaling with the hydrophobicity of the sticker.^46^ Of course, the intermediate time range can be interesting for a further understanding of the relation between rheological properties and mesoscopic structure. To narrow the time scale gap between simulation and experiment, one can prolong the atomistic simulations to cover time scales of 10^–6^ up to 10^–4^ s (1 MHz to 0.1 kHz). With coarse-grained simulations, even longer time scales are accessible. Other concerns are the limited size of the simulation box, which might introduce a spurious periodicity in the system, the higher peptide concentration in the simulation compared to the experiment, and the water model used. Using a 4-site water model^47^ or a polarizable water model,^48^ will yield a more realistic representation of the diffusive dynamics of the coiled-coil dimers and of the water structure in the solvation shell of the peptides.

Nonetheless, our study reveals that modifying the chromophore label is a synthetically simple strategy to shape the interactions between coiled-coil dimers and thereby tune the viscoelastic properties of the peptide hydrogel. By adding a chromophore label, a hydrophobic group is introduced at the N-terminus, and the solvent-exposed amino group is shifted away from the peptide backbone. These two effects counterbalance each other. The presence of the hydrophobic group increases the hydrophobicity of the N-terminus, while the shift of the amino group weakens the intramolecular salt bridge, thus increasing the solvent exposure of the amino group and a nearby glutamate residue. The third effect of the chromophore label is sterically changing the salt network, which stabilizes the interface between two coiled-coil dimers. By shifting the amino group away from the peptide backbone, the interface is sterically less crowded, which increases oligomer size and stability in oaba-hFF03. However, when placing the amino group in para-position, the amino group cannot as easily be oriented toward the interface, and as a consequence, oligomer size and stability are lower in paba-hFF03 than in oaba-hFF03.

In conclusion, the three parameters of the chromophore label, size of the aromatic system, distance between the carboxyl group and amino group, orientation of the amino group relative to the carboxyl group, open up a design strategy to control the viscoelastic properties of hFF03 peptide hydrogels.

## Supporting Information Available

Detailed explanation on how to calculate the local velocity auto correlation function and diffusion coefficient, oscillatory rheology result of no-hFF03, snapshot of a self-assembled fibril network for oaba-hFF03 and no-hFF03, and numerical overview of all counted salt-bridge interaction for all self-assembly simulations (PDF)
