# Supplementary material for: How Chromophore Labels Shape the Structure and Dynamics of a Peptide Hydrogel
Source: Biomacromolecules. 2024 Jan 30;25(2):1262–73. doi: 10.1021/acs.biomac.3c01225 (PMC10865361; doi:10.1021/acs.biomac.3c01225)
Supplement: Supplementary file 1 — bm3c01225_si_001.pdf [file bm3c01225_si_001.pdf]

Supplementing Information: How chromophore labels shape the structure and dynamics of a peptide hydrogel

Frederick Heinz, Jonas Proksch, Robert F. Schmidt, Beate Koksch, Michael Gradzielski, and Bettina G. Keller

(Dated: 15 December 2023)

I. CALCULATION OF THE LOCAL DIFFUSION COEFFICIENT

Algorithm 1 summarizes our approach to computing the local diffusion coefficient

Algorithm 1 Local Diffusion Coefficient

```
Discretize the radial distance into bins
for each H2O in a trajectory do
    Calculate v_COM                                  ▷ COM velocity
    Calculate q_COM                                  ▷ COM position
    Cut trajectory into 5 ps long segments
    for each trajectory segment do ▷ calculation of VACF
        Assign trajectory segment to radial distance bin using q_COM
        calculate VACF via FFT (including zero padding)
    end for
    Add the VACF in each distance bin and normalize
end for
for each bin do
    Normalize by the number of VACFs.
    Numerically integrate VACF (eq. 4)
end for
```

The FFT is carried out for each spatial direction of $\vec{v}_{COM}$ separately. To calculate the VACF via FFT, proceed as follows:

- Zero padding[1]
- $FFT(\bar{v}_{COM})$
- Multiply by its complex conjugate
- irFFT (real part of inverse FFT)
- Reverse zero padding
- Divide by number of frames. (This step depends on the used FFT implementation)

Then average over all three dimensions.

[1] S. Hilbert, "FFT Zero Padding — BitWeenie," https://www.bitweenie.com/listings/fft-zero-padding/ (2013).

II. ADDITIONAL FIGURES

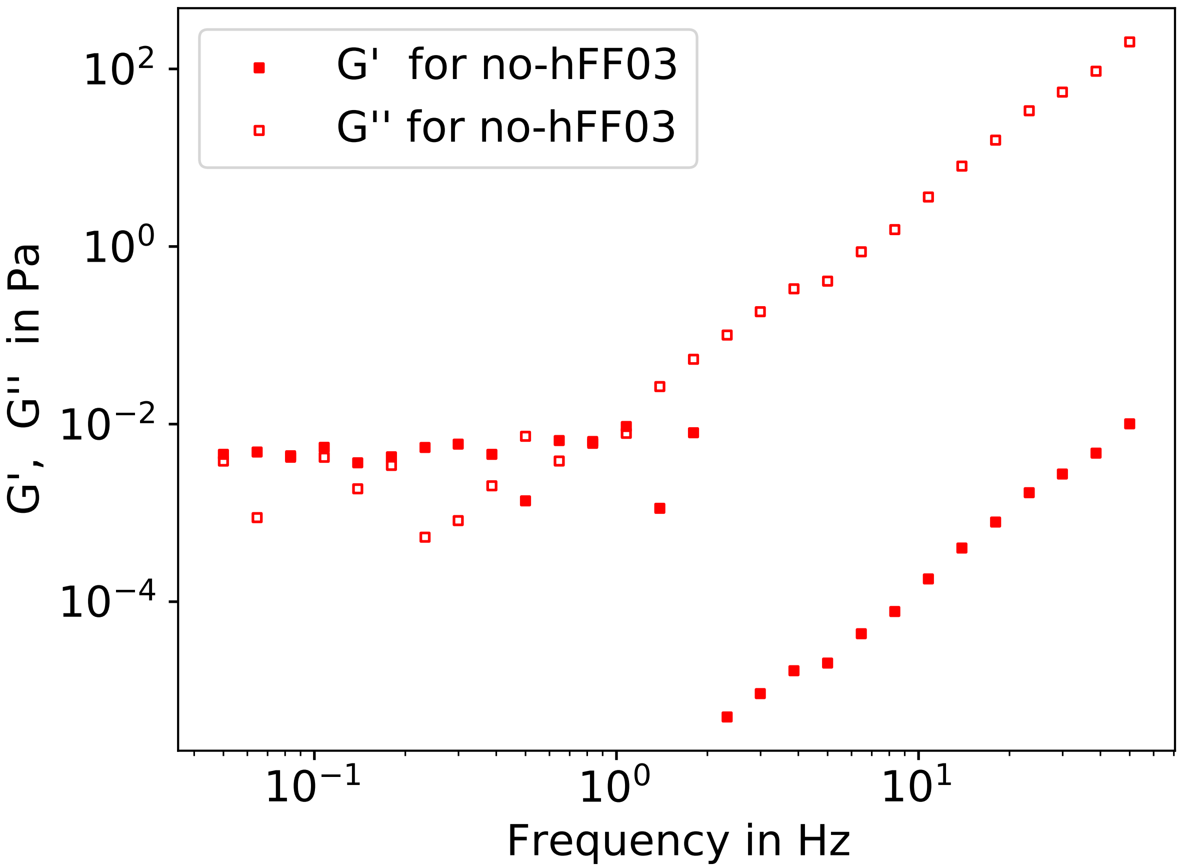

FIG. 1. Frequency sweep measured through oscillatory shear experiment of no-hFF03. The shown features are a result of measurement artefacts and can be attributed to effects of instrument and fluid inertia.

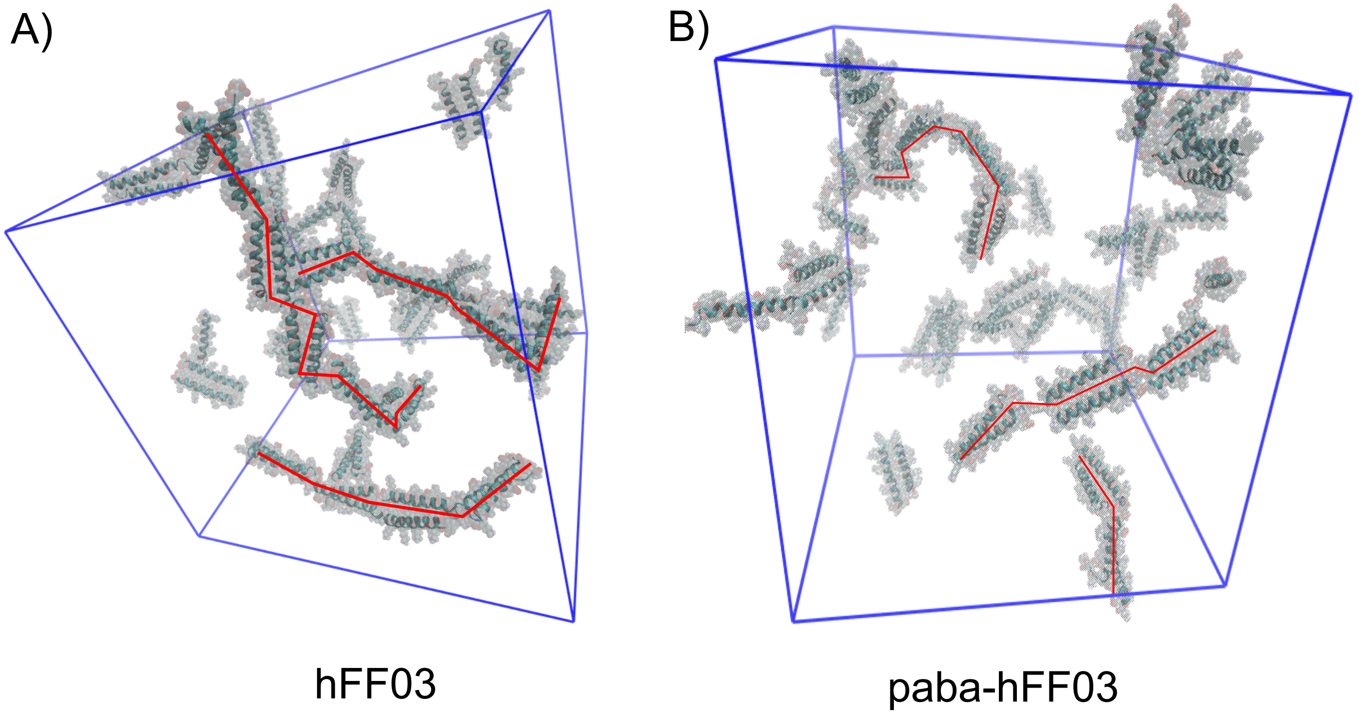

FIG. 2. Snapshots of no-hFF03 (A) and paba-hFF03 (B) in explicit water after 50 ns simulation (4 wt%, starting structure with randomly placed coiled-coils). Red lines highlight self assembled oligomers.

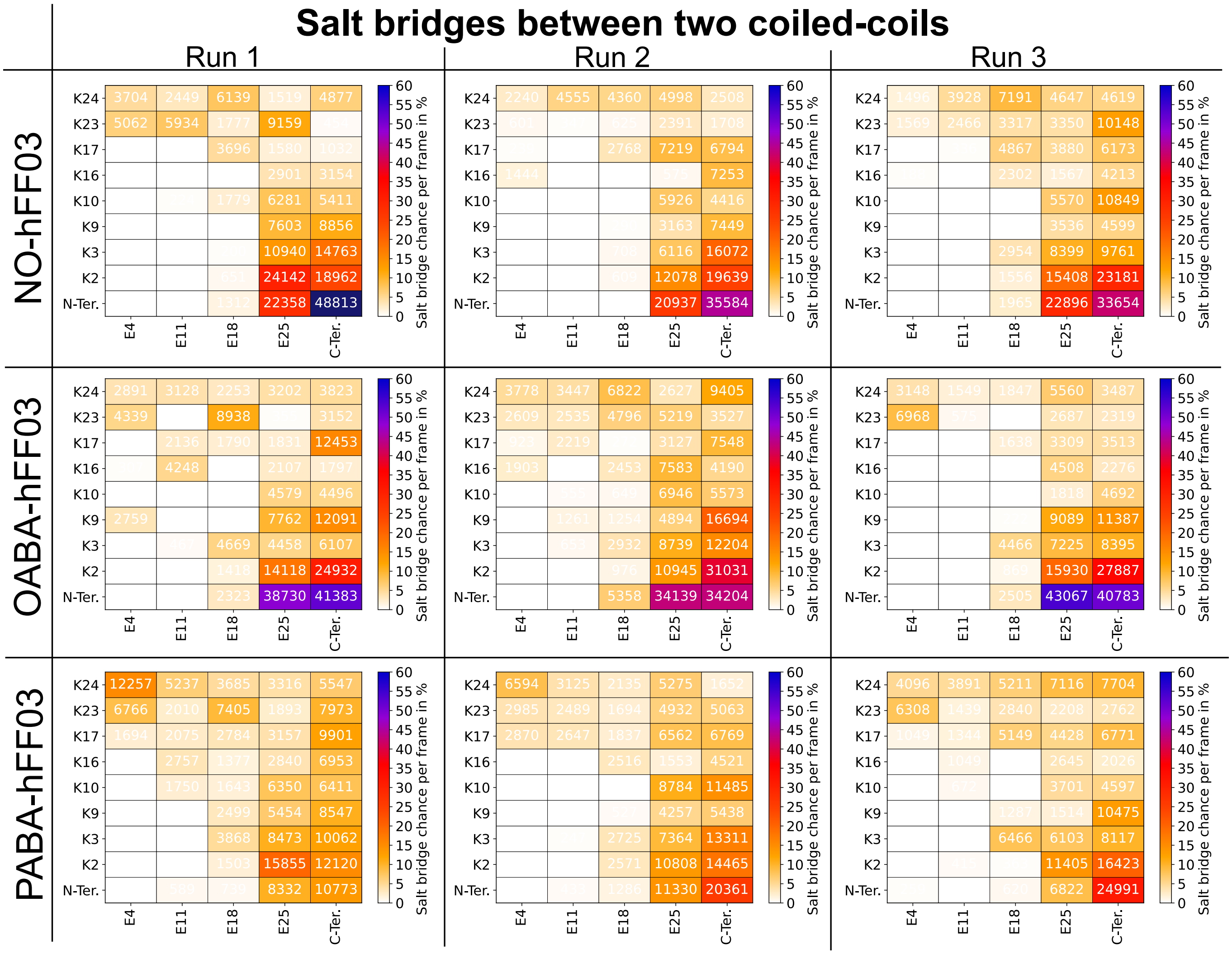

FIG. 3. Populations of salt-bridges between of coiled-coil dimers. These salt-bridges stabilize the fibril. The numbers represents in the number of frames in which the salt bridge occurs in the time interval 100 ns to 150 ns of the MD simulation (total of 25.000 frames).

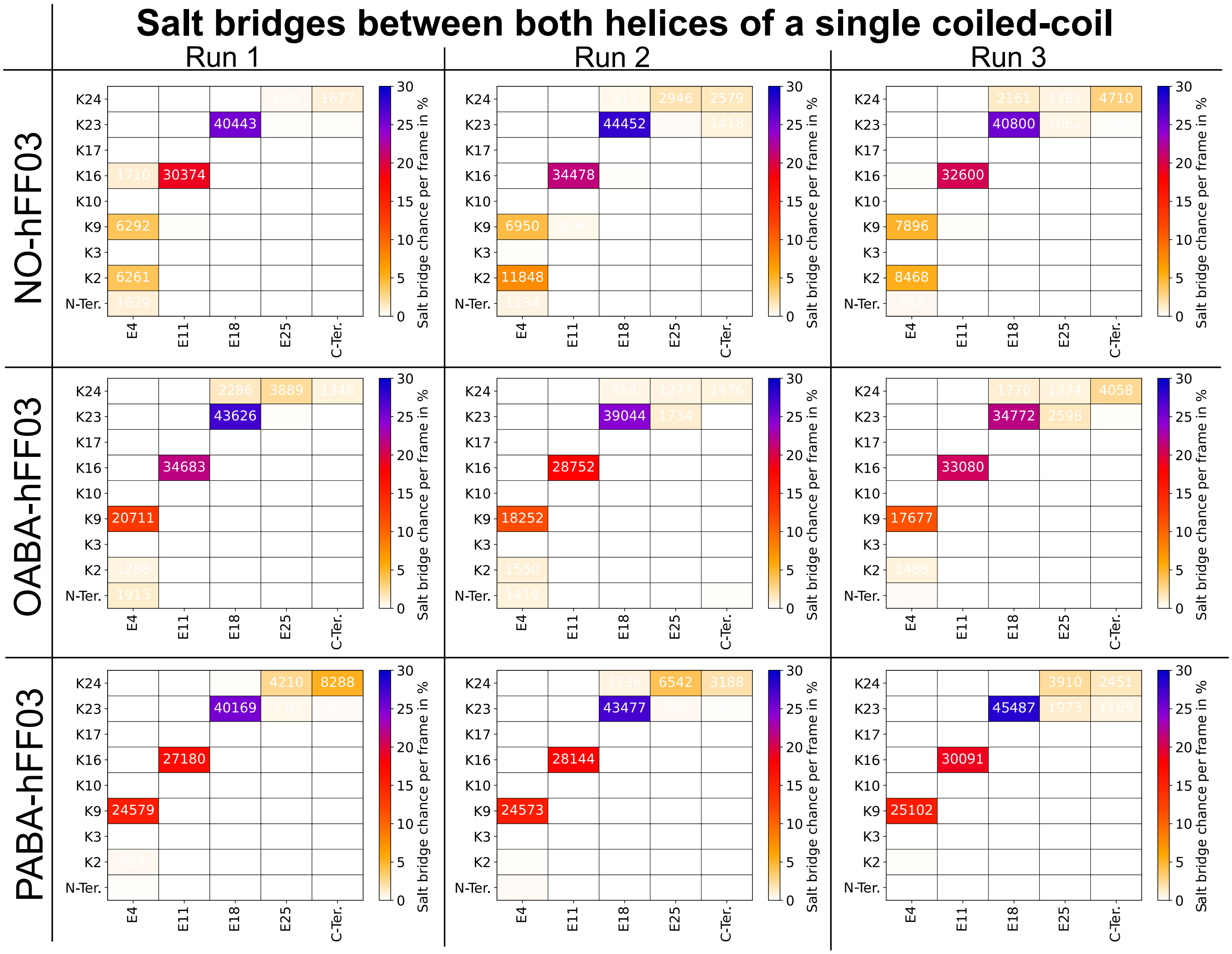

FIG. 4. Populations of salt-bridges between of α-helices within a coiled-coil dimer. These salt-bridges stabilize the dimer in addition to the leucine zipper motif. The numbers represents in the number of frames in which the salt bridge occurs in the time interaval 100 ns to 150 ns of the MD simulation (total of 25.000 frames).

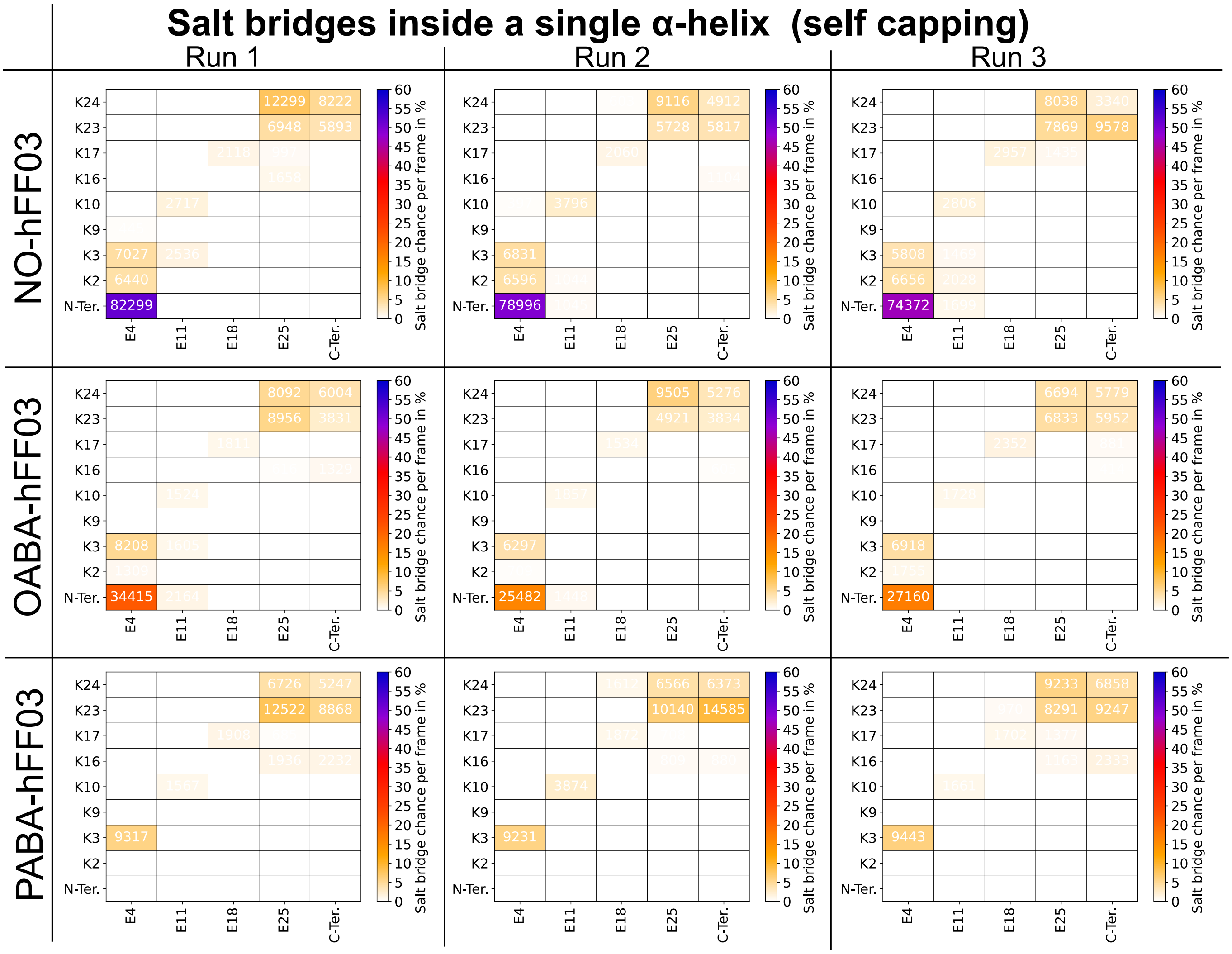

FIG. 5. Populations of salt-bridges within a peptide chain. These salt-bridges stabilize the self-capping of the N-terminus. The numbers represents in the number of frames in which the salt bridge occurs in the time interaval 100 ns to 150 ns of the MD simulation (total of 25.000 frames).
